# Blood Eosinophils and Exhaled Nitric Oxide: Surrogate Biomarkers of Airway Eosinophilia in Stable COPD and Exacerbation

**DOI:** 10.3390/biomedicines10092128

**Published:** 2022-08-30

**Authors:** Balazs Antus, Imre Barta

**Affiliations:** 1 Department of Pathophysiology, National Koranyi Institute of Pulmology, Koranyi Frigyes Ut 1, 1121 Budapest, Hungary; 2 Department of Pulmonology, National Koranyi Institute of Pulmology, Koranyi Frigyes Ut 1, 1121 Budapest, Hungary

**Keywords:** eosinophilic, inflammation, lung, non-invasive, phenotype, responsiveness, sensitivity, specificity, sputum, steroid

## Abstract

In recent years, tremendous efforts have been devoted to characterizing the inflammatory processes in chronic obstructive pulmonary disease (COPD) in order to provide more personalized treatment for COPD patients. While it has proved difficult to identify COPD-specific inflammatory pathways, the distinction between eosinophilic and non-eosinophilic airway inflammation has gained clinical relevance. Evidence has shown that sputum eosinophil counts are increased in a subset of COPD patients and that these patients are more responsive to oral or inhaled corticosteroid therapy. Due to feasibility issues associated with sputum cell profiling in daily clinical practice, peripheral blood eosinophil counts and fractional exhaled nitric oxide levels have been evaluated as surrogate biomarkers for assessing the extent of airway eosinophilia in COPD patients, both in stable disease and acute exacerbations. The diagnostic value of these markers is not equivalent and depends heavily on the patient’s condition at the time of sample collection. Additionally, the sensitivity and specificity of these tests may be influenced by the patient’s maintenance treatment. Overall, eosinophilic COPD may represent a distinct disease phenotype that needs to be further investigated in terms of prognosis and treatment outcomes.

## 1. Introduction

Chronic obstructive pulmonary disease (COPD) is an inflammatory lung disease characterized by increased numbers of macrophages, neutrophils and T cells, and enhanced release of inflammatory mediators including cytokines, chemokines, growth factors, oxidants and vasoactive agents [1]. There is evidence that airway inflammation is further increased in acute exacerbations of COPD (AECOPD) defined as an acute, transient deterioration in patients’ symptoms and lung function [2,3]. Nonetheless, COPD is heterogeneous in terms of symptoms and underlying inflammatory processes, and the patient’s response to treatment, particularly inhaled corticosteroids (ICS), is variable.

In recent years, tremendous efforts have been made to elucidate the inflammatory pathways in COPD to better understand the molecular mechanisms underlying the disease and to lay the foundation for the use of biological agents targeting specific inflammatory pathways [4,5]. However, the identification of distinct inflammatory endotypes in COPD patients has been challenging, and the relationship between inflammatory mechanisms and clinical manifestations of the disease remained uncertain in most cases [6]. Likewise, although different phenotypes of AECOPD have been characterized, this has had little impact on the treatment protocols used in the routine clinical setting for patients with AECOPD [7]. Rather than defining COPD-specific inflammatory pathways, several studies have documented increased sputum eosinophil numbers in a subset of COPD patients; both in stable disease [8] and in exacerbations [9]. Studies have also shown that patients in this group respond favorably to corticosteroid therapy [8,10,11,12], have a reduced airway bacterial burden [13], and exhibit more pronounced improvement following treatment for AECOPD [14], suggesting that they may have a distinct clinical phenotype [15]. Therefore, the distinction between eosinophilic and non-eosinophilic airway inflammation is of clinical importance.

Nonetheless, such a distinction has yet to be implemented in everyday clinical practice, where, in most cases, neither sputum induction nor its processing and evaluation are feasible. In this review, we will focus on two established biomarkers, fractional exhaled nitric oxide (FENO) levels [16,17] and peripheral blood eosinophil count [18,19], and summarize the current evidence on how these measures can be used to provide sensitive and specific estimates of airway eosinophilia in patients with stable COPD and AECOPD.

## 2. Eosinophilic Airway Inflammation in COPD

It is well established that chronic airway inflammation plays a central role in the pathophysiology of COPD. Nonetheless, the airway inflammation in COPD is heterogeneous: the most common inflammatory phenotype is neutrophil-associated COPD with inflammasome, Th1 and Th17 activation, while in a minority of patients increased eosinophilic airway inflammation with increased Th2-transcriptome signature can be observed [20]. Exposure to cigarette smoke induces the recruitment of inflammatory cells into the airways and stimulates innate and adaptive immune responses [21]. Inflammatory changes occur both in the proximal and distal airways and in the lung parenchyma, where they manifest in the increased numbers of inflammatory cells, particularly neutrophil granulocytes, macrophages and CD8+ lymphocytes [22,23]. Activated leukocytes release several inflammatory mediators, i.e., cytokines, chemokines, growth factors, oxidants, proteases, and vasoactive and bronchoconstrictor agents, which in turn may further promote inflammatory cell migration into the airways and contribute to the development of the pathomorphological changes typical for COPD [24]. Inflammation is also present in the pulmonary artery wall and may be involved in the development of COPD-associated co-morbidities as well.

There is increasing evidence that oxidative stress is the main driving mechanism for the development of airway inflammation in COPD [25,26]. It has been proposed that apart from the burden of inhaled oxidants and reactive oxygen species generated in the airways, depletion of antioxidants may also be partly responsible for the oxidant/antioxidant imbalance that characterizes this condition.

Although the predominant feature of COPD is neutrophilic inflammation, there is evidence indicating that a subset of patients (10–30%) have increased numbers of eosinophils in the airways [8], as is typically seen in patients with asthma. The definition of sputum eosinophilia in COPD is variable in the literature, with most studies defining it as 2–3% of sputum leukocytes. It is well known that eosinophils are inflammatory cells consisting of bi-lobed nuclei and large acidophilic cytoplasmic granules, and are produced in bone marrow from CD34+ myeloid progenitor cells [27]. Upon maturation, eosinophils enter the systemic circulation and migrate primarily to the gastrointestinal tract and thymus. The circulating eosinophils are recruited into the airways by immunoregulatory cells and chemokines. The recruitment of eosinophils to the airways is under the control of specific chemokines and their cognate receptors and typically occurs in the context of a Th2 inflammatory response [1,22]. It is believed that the quality and the activation state of eosinophils may be more important than the absolute eosinophil number in the context of the inflammatory response [28].

From a clinical perspective, studies have shown that compared to the neutrophil predominant group, the eosinophilic subgroup of COPD patients respond better to inhaled short-acting β_2_-agonists [29], benefit more from short-course inhaled [8] or oral corticosteroid therapy [10,11,12], and have a lower airway bacterial burden [13] as mentioned above. The number of eosinophils in the airways does not appear to correlate strongly with disease progression as determined by GOLD staging of the disease [30]. However, the SPIROMICS study investigating a large and well-characterized cohort of COPD patients has shown that patients with elevated sputum eosinophil counts have worse lung function and more pronounced emphysema than those with low sputum eosinophil counts [31]. Moreover, the high eosinophil count group had more frequent episodes of AECOPD requiring corticosteroid treatment than the low eosinophil group. Additionally, Siva et al. demonstrated that a COPD management strategy with the additional aim of reducing eosinophilic airway inflammation in stable COPD patients was associated with a reduction in subsequent AECOPD in these subjects [32].

Although AECOPD is typically associated with increased neutrophilic inflammation in the airways, eosinophilia may also be present in the sputum of some patients with AECOPD [9]. Our data indicate that the clinical characteristics of eosinophilic and non-eosinophilic AECOPD patients are not alike; we have recently observed less purulent sputum and lower C-reactive protein (CRP) levels in eosinophilic subjects compared to non-eosinophilic AECOPD patients [33]. In terms of systemic inflammatory marker levels, similar findings were obtained by Csoma and co-workers as well [34]. These findings are consistent with the general view that eosinophilic exacerbations are triggered by viral infections, whereas exacerbations of bacterial origin may be associated with neutrophilic inflammation and elevation in systemic inflammatory markers [13,35]. In addition, we have demonstrated that AECOPD patients with sputum eosinophilia exhibit a more pronounced improvement in airflow limitation following treatment [14]. Overall, these data suggest that eosinophilic COPD has a distinct disease phenotype in both clinically stable states and AECOPD.

## 3. Assessment of Eosinophilic Airway Inflammation Using FENO

In recent years, FENO has been used extensively as a surrogate biomarker to determine and quantify the extent of airway eosinophilia in various respiratory diseases including COPD [16,17]. From a technological point of view, chemiluminescence-based analysis is the gold standard method for FENO measurement [36]. Although chemiluminescence analyzers are fast-reacting, highly sensitive and specific for nitric oxide gas, their size, cost and frequent need for calibration limit their penetration into routine clinical practice. To overcome these limitations, electrochemical sensors suitable for the detection of FENO in the exhaled breath have been developed and incorporated into handheld measuring devices. Data from our [37] and other [38,39] laboratories demonstrated that FENO values measured with such handheld devices are highly reproducible and in good agreement with those obtained with chemiluminescence analyzers, making them suitable for use in clinical practice.

The rationale for using FENO in the assessment and management of various respiratory diseases is based on two key factors: first, there is a highly significant relationship between FENO levels and the extent of eosinophilia in airway specimens such as induced sputum, bronchoalveolar lavage and biopsy materials; second, there is an equally important relationship between eosinophilic airway inflammation and steroid responsiveness [40]. Although these associations have been established primarily in patients with asthma, apparently they also apply to patients with other respiratory pathologies.

When looking at the stable COPD population as a whole, FENO levels are similar [41] or only slightly increased [42] compared to those in healthy controls, as demonstrated more than two decades before. This is not unexpected since, as mentioned above, COPD is typically associated with neutrophilic airway inflammation, and eosinophilia, which is associated with elevated FENO levels, is present only in a minority of patients. Early studies [43] suggested that FENO may also be a marker of disease severity in COPD; however, subsequent studies have not shown a consistent association between FENO levels and lung function impairment in COPD patients. More recently, extended NO analysis, i.e., the measurement of alveolar and bronchial NO, has been suggested as a more useful method to monitor nitrative stress at different anatomical sites within the airways in stable and exacerbated COPD patients [44].

Despite the correlation between FENO levels and the number of eosinophils in the airways, the predictive accuracy of FENO measurement for eosinophilia appears to be variable and often only modest. For example, Chou et al. found a sensitivity of 62% and a specificity of 71% for FENO measurement in identifying COPD patients with eosinophilic airway inflammation [45]. In our recent study, similar sensitivity (63%) but higher specificity (91%) values were observed when the cut-off value for sputum eosinophilia was set at 3% [33]. Importantly, the negative predictive value (NPV) of the test was high (93%) indicating that the clinical relevance of using FENO lies in its ability to reliably identify non-eosinophilic subjects. FENO alone [46] or in combination with blood eosinophil count [47] has been implicated in the differential diagnosis of asthma-COPD overlap (ACO) and COPD as well. However, sputum analysis was not performed in these studies. Finally, it should be noted that FENO could also be a marker of steroid responsiveness in patients with COPD; however, its usefulness is limited to predicting an increase in forced expiratory volume in one second (FEV_1_) following a short course of oral corticosteroid treatment [48].

Concerning exacerbation, FENO levels are mostly elevated at the onset of AECOPD, while treatment of exacerbation or recovery from exacerbation leads to a decrease in FENO concentrations [49,50]. Additionally, we have demonstrated that FENO levels measured during hospitalization for AECOPD correlate closely with improvements in airflow limitation, as reflected by an increase in FEV_1_ after treatment [51]. Moreover, patients with higher FENO levels at the onset of AECOPD were typically discharged earlier, as a better functional response is associated with faster clinical recovery.

In a follow-up study, we further showed that there is a significant association between FENO level and the percentage or number of eosinophils in the sputum of AECOPD patients, and that FENO is a strong predictor of sputum eosinophilia (>3%) in AECOPD (area under the receiver operating characteristic curve [ROC AUC]: 0.89) [14]. Overall, these data suggest that measuring FENO may assist in selecting patients with AECOPD who have sputum eosinophilia and are potentially more responsive to treatment. Although we could not determine what was responsible for the better treatment outcome, we hypothesized that corticosteroids had a predominant effect, as suggested by several studies in stable COPD patients [10,11,12]. In contrast, in patients with clinically stable COPD, FENO measurement has only limited diagnostic value, as noted above. The current Global Initiative for Chronic Obstructive Lung Disease (GOLD) guideline also does not recommend the use of FENO measurement in the management of COPD patients [52].

There may be an association between FENO levels and the frequency of exacerbations in COPD. We found that COPD patients with low FENO levels during acute exacerbations were more susceptible to developing severe AECOPD subsequently, while those with elevated FENO levels during exacerbation were less likely to be hospitalized for AECOPD [53]. Although this was only a retrospective analysis of a relatively small number of patients, it is tempting to speculate that FENO measurement may also serve as a predictive tool for the incidence of exacerbations associated with hospitalization. We believe that is a promising area for further prospective investigation in the future.

Cigarette smoking lowers FENO levels in COPD patients and is widely considered an important confounder in FENO measurements that should be carefully assessed in all studies [16,17]. For example, Gao et al. recently reported a lower predictive value for FENO (ROC AUC: 0.73) than established by many other research groups in the evaluation of sputum eosinophilia in AECOPD [54]. However, in this study, a significant proportion of participants were active smokers, so the smoking status of patients could affect these results.

Another controversial issue that has been addressed in several recent studies is the effect of ICS therapy on FENO levels. Early studies indicated that ICS treatment may affect FENO levels [55,56], although there were also studies that showed no significant effect [57]. In a recent meta-analysis of only randomized clinical trials or two-arm controlled prospective studies, the authors concluded that FENO levels are significantly reduced with ICS treatment in ex-smokers with COPD, while the effect in smokers is less clear and needs further investigation [58]. Again, this suggests that the ICS status of the patient has to be considered carefully in all clinical studies.

## 4. Assessment of Eosinophilic Airway Inflammation Using Blood Eosinophils

Although the relevance of blood eosinophils in the management of COPD patients, particularly in guiding ICS therapy to prevent exacerbation, has been extensively investigated in previous years, the evidence on the relationship between local (airway) and systemic (blood) eosinophilia remains controversial. Negewo et al., for example, found that blood eosinophil counts predict sputum eosinophilia with relatively high accuracy in patients with stable COPD [59], while investigators of the SPIROMICS cohort concluded that blood eosinophilia alone is not a reliable marker of airway eosinophilia (or the eosinophilic COPD phenotype) despite the highly significant correlation between the two measures [31]. In this context, we found that blood eosinophil counts are a good indicator of airway eosinophilia in patients with stable COPD, but not in patients with ongoing exacerbation where the sensitivity of the test is poor (20–40%) [33]. This suggests that the diagnostic value of blood eosinophils depends on the patient’s condition at the time of sample collection, as eosinophilic airway inflammation in AECOPD does not necessarily lead to systemic eosinophilia.

Despite these uncertainties, several post hoc analyses of randomized controlled trials demonstrate that blood eosinophil count is an independent predictor of response to ICS in patients with severe or very severe COPD and a history of exacerbations [19,28,60]. In line with this view, the current GOLD document provides therapeutic recommendations for blood eosinophil counts and advises that thresholds of <100 cells/μL and ≥300 cells/μL can be used to identify patients with a low and high likelihood of benefiting from ICS-containing therapy, respectively [52]. This is clinically very important, as it allows the blood eosinophil count to be used as a biomarker to maximize the benefit/risk ratio of ICS treatment and to move towards a personalized medicine approach in the management of COPD.

However, some investigators also emphasize that blood eosinophil counts should be considered as a continuum and evaluated in the context of other risk factors for exacerbations, and cut-off values should not be treated as explicit [61]. In the above-mentioned analysis [60], the authors modeled the number of eosinophils as a continuous variable to identify the characteristics that determine both the risk of exacerbation and the clinical response to ICS in patients with COPD. Following this approach, the data from the IMPACT trial were also analyzed, and the investigators found that the response to ICS-containing therapy was modulated by blood eosinophil count, namely that the benefits of ICS-containing treatments in terms of reducing the rate of moderate/severe and severe exacerbations increased with increasing blood eosinophil count [62]. This association was further adjusted for the smoking status of the patients; former smokers showed a greater benefit at all blood eosinophil counts than current smokers. These data emphasize the importance of smoking cessation with the observation that current smokers with lower blood eosinophil count (<200 cells/μL) did not appear to benefit from ICS-containing triple therapy over long-acting muscarinic antagonist (LAMA) plus long-acting β_2_-agonist (LABA) therapy, whereas former smokers showed benefits across the whole blood eosinophil continuum.

There are conflicting results in the literature on the effects of ICS on blood eosinophils: some investigators have found no difference between ICS users and non-users [13], while others have found reduced eosinophil counts as a result of ICS treatment [8]. A recent retrospective analysis of a clinical trial comparing the effects of various bronchodilators concluded that ICS has only a small effect on peripheral blood eosinophils in steroid-naïve COPD patients [63]. Furthermore, in a post hoc analysis of the ISOLDE trial, the authors found that changes in blood eosinophil count after ICS administration predicted clinical response to ICS therapy in patients with moderate-to-severe COPD at risk of exacerbation [64]. Interestingly, an increase in the number of exacerbations and an accelerated lung function decline was documented in the 20% of patients whose blood eosinophil levels increased after ICS administration.

It has been established that the stability of blood eosinophil count and the reproducibility of its measurement may be important confounding factors that may limit the use of this biomarker in the management of COPD patients. The issue has been intensively investigated in recent years. Landis et al., for example, explored the reproducibility of blood eosinophil counts in a large cohort of stable COPD patients over 1 year and concluded that reproducibility was good, although eosinophil counts were variable in a subset of patients [65]. Negewo et al. also assessed the stability of blood eosinophil counts in patients with stable COPD over a median 28-day period and found good agreement between the two measurements [59]. Oshagbemi and co-workers also conducted a study to estimate the reproducibility of eosinophil counts in general practice and concluded that stability after 6 months was around 85% [66]. Two years later, this rate declined to 62%, with a further progressive decline in the subsequent years.

However, other studies have found lower reproducibility of blood eosinophils in COPD patients. For example, in the COSYCONET study, an analysis of a subgroup of COPD patients over routine examinations at 0, 6 and 18 months revealed that 26% of patients were persistently non-eosinophilic, while only 5% remained eosinophilic at all three visits [67]. Similarly, when blood (or sputum) eosinophilia was defined as ≥2% in a retrospective analysis of the ECLIPSE study, only 37.4% of COPD patients exhibited stable blood eosinophilia [68]. Another important question is whether the variability of blood eosinophil count results in the crossing of a given threshold that would assign an individual to a different ICS response category. Investigating this point, Southworth et al. reported that over repeated eosinophil measurements (6 months and >2 years) the majority (>86%) of patients remained in the same category, even at a threshold as low as 150 cells/μL [69]. Nonetheless, higher blood eosinophil counts are associated with higher variability, as indicated by this [69] and other [70] studies.

Maintenance therapy of patients, in particular, ICS-containing treatments, may impact the stability of blood eosinophil count, as they reduce the number of eosinophils, which in turn may improve the reproducibility. However, a variety of other factors such as atopy, co-morbidities, medications, or infections may also affect the reproducibility of eosinophil counts in the blood, especially in the long term.

There is evidence that patients with elevated eosinophil counts during exacerbations respond more favorably to systemic corticosteroid therapy [71,72] and have lower rates of early treatment failure [73]. Whether increased eosinophil counts during AECOPD are associated with recurrent exacerbations is unclear, as studies investigating the relationship between eosinophil counts or ratios and the frequency of exacerbations have revealed conflicting results [73,74,75]. A recent prospective study that addressed this question in a cohort of patients hospitalized for severe AECOPD has indicated that patients with eosinophilic exacerbations did not have an increased risk of early, moderate or severe relapses [34].

Nonetheless, it appears that the blood eosinophil count in patients hospitalized with severe AECOPD is of clinical relevance. In support of this concept, MacDonald and co-workers recently reported that patients with low eosinophil counts (<50 cells/μL) at the onset of AECOPD were more likely to have pulmonary infection, longer hospital stays and lower 12-month survival than those with elevated eosinophil counts (>150 cells/μL) [76].

Finally, it should be noted that in addition to their role as biomarkers for ICS responsiveness, blood eosinophils may also have relevance in predicting future COPD exacerbations. However, a recent study that examined pooled data from 11 clinical trials found no clinically important association between baseline blood eosinophil count and exacerbation rate, so these results do not support the use of blood eosinophils to predict exacerbation risk [77].

## 5. Is It Worth Combining the Measurement of FENO and Blood Eosinophil Count?

Since FENO and blood eosinophils are regulated by different inflammatory pathways, it is reasonable to consider whether simultaneous measurement of the two surrogate markers would improve the chances of identifying patients with eosinophilic airway inflammation.

Nevertheless, there are only a few reports that explore this possibility. Colak and colleagues analyzed data from the Copenhagen General Population Study of 4677 individuals with chronic respiratory symptoms after dividing them into six groups based on previous respiratory disease diagnoses, and found that the levels of the two biomarkers were not predictive of airway disease type, and combining them did not provide any benefit [78]. In our experiments, the combination of FENO and blood eosinophil counts resulted in increased sensitivity but decreased specificity and PPV in predicting airway eosinophilia in stable COPD compared to blood eosinophil count alone [33]. Similarly, in AECOPD, the combination of the two measurements did not result in a significant improvement in diagnostic accuracy compared to FENO alone. However, as mentioned before, the combined measurement may have potential in the diagnosis of ACO by improving the overall sensitivity of the test to above 90% [79].

## 6. Conclusions

COPD is a chronic respiratory disease characterized by persistent respiratory symptoms, impaired quality of life, chronic airway inflammation, and, in most cases, a progressive decline in lung function. There is evidence that a minority of patients have an eosinophilic rather than a neutrophilic type of airway inflammation, and these patients have a distinct disease phenotype. FENO and blood eosinophil count are both useful surrogate markers of airway eosinophilia in COPD, but, as summarized in Table 1, there are advantages and disadvantages to each test.

**Figure 1:**
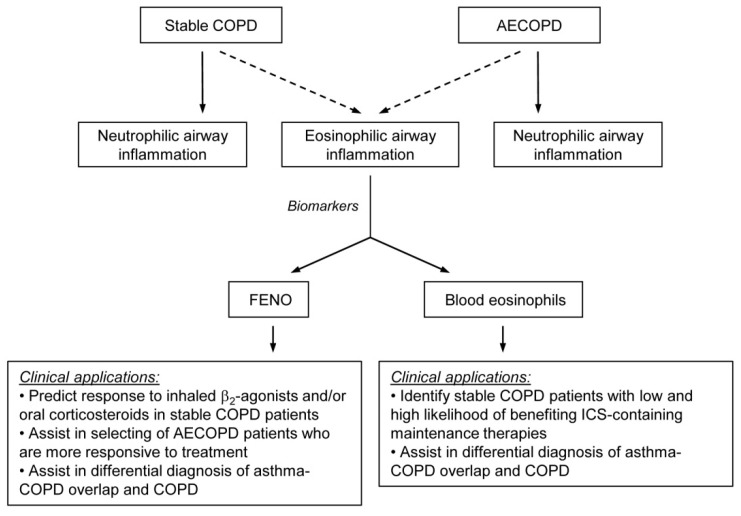
Schematic representation of the utility of fractional exhaled nitric oxide (FENO) levels and peripheral blood eosinophil counts as surrogate biomarkers for assessing eosinophilic airway inflammation in stable COPD and acute exacerbations (AECOPD). ICS: inhaled corticosteroid.

With regard to the clinical applicability of FENO and blood eosinophil measurements (Figure 1), it depends, at least in part, on the condition of the patient. The blood eosinophil count can help clinicians identify patients with stable COPD who would benefit most from maintenance therapy with ICS, while FENO can be valuable in selecting patients with AECOPD who are more responsive to acute treatment. The literature is equivocal on whether the percentage or the absolute eosinophil count is more predictive for airway eosinophilia. The GOLD document recommends using the absolute eosinophil count with thresholds of 100 and 300 cells/μL; therefore, clinicians have to rely on this parameter. Furthermore, FENO may aid in predicting response to inhaled β_2_-agonists and/or oral corticosteroids in stable COPD. Both markers may assist the clinician in the differential diagnosis of ACO and COPD.

The concept of treatable traits is a precision medicine approach that has recently been proposed for the management of obstructive airway diseases, including COPD [80,81]. It should be considered as a model of care in which the patient undergoes a multidimensional assessment to identify clinically relevant and treatable problems (traits). As airway eosinophilia is one of the key treatable traits in this model, measurement of both FENO levels and blood eosinophil counts could be useful in the management of COPD patients based on clinical aspects summarized in Figure 1. Nonetheless, the current GOLD document favors eosinophil count over FENO in clinical decision-making; thus, at present, the measurement of blood eosinophil levels is primarily aimed at guiding the use of ICS-containing therapies (ICS/LABA or ICS/LABA/LAMA) to prevent exacerbations in patients with multiple exacerbations.

In conclusion, further studies are needed to explore the clinical benefit of measuring these biomarkers in the management of patients with COPD. In particular, studies that include sputum analysis to provide reliable information on the local inflammatory cell profile of the airways would have real clinical relevance.

## Figures and Tables

**Table 1:** Advantages and disadvantages of measuring fractional exhaled nitric oxide (FENO) levels and peripheral blood eosinophil counts in patients with COPD.

	FENO	Blood Eosinophils
Difficulty for patients	requires cooperation	simple
Availability of test	sporadic	universal
Burden for patients	non-invasive	semi-invasive
Methodology	standardized	standardized
Results	immediate	within hours
Reproducibility	good	good ^1^
Follow-up measurements	easy	easy
Specificity for airway inflammation	high	low
Confounding factors	smoking	co-morbidities

^1^ poor for a subset of patients.

## Data Availability

Not applicable.
